# A comparative study of enzyme initiators for crosslinking phenol-functionalized hydrogels for cell encapsulation

**DOI:** 10.1186/s40824-016-0077-z

**Published:** 2016-10-05

**Authors:** Justine J. Roberts, Pratibha Naudiyal, Khoon S. Lim, Laura A. Poole-Warren, Penny J. Martens

**Affiliations:** 1 Graduate School of Biomedical Engineering, UNSW Australia, Sydney, 2052 NSW Australia; 2 Christchurch Regenerative Medicine and Tissue Engineering (CReaTE) Group, Department of Orthopaedic Surgery and Musculoskeletal Medicine, University of Otago Christchurch, Christchurch, 8011 New Zealand

**Keywords:** Oxidative enzyme, Hydrogel, Phenol-crosslinking, Cell encapsulation

## Abstract

**Background:**

Dityrosine crosslinking in proteins is a bioinspired method of forming hydrogels. This study compares oxidative enzyme initiators for their relative crosslinking efficiency and cytocompatibility using the same phenol group and the same material platform. Four common enzyme and enzyme-like oxidative initiators were probed for resulting material properties and cell viability post-encapsulation.

**Results:**

All four initiators can be used to form phenol-crosslinked hydrogels, however gelation rates are dependent on enzyme type, concentration, and the oxidant. Horseradish peroxidase (HRP) or hematin with hydrogen peroxide led to a more rapid poly (vinyl alcohol)-tyramine (PVA-Tyr) polymerization (10–60 min) because a high oxidant concentration was dissolved within the macromer solution at the onset of crosslinking, whereas laccase and tyrosinase require oxygen diffusion to crosslink phenol residues and therefore took longer to gel (2.5+ hours). The use of hydrogen peroxide as an oxidant reduced cell viability immediately post-encapsulation. Laccase- and tyrosinase-mediated encapsulation of cells resulted in higher cell viability immediately post-encapsulation and significantly higher cell proliferation after one week of culture.

**Conclusions:**

Overall this study demonstrates that HRP/H_2_O_2_, hematin/H_2_O_2_, laccase, and tyrosinase can create injectable, *in situ* phenol-crosslinked hydrogels, however oxidant type and concentration are critical parameters to assess when phenol crosslinking hydrogels for cell-based applications.

**Electronic supplementary material:**

The online version of this article (doi:10.1186/s40824-016-0077-z) contains supplementary material, which is available to authorized users.

## Background

Hydrogels have great potential for biomedical applications and have been extensively explored for drug delivery, cell encapsulation and tissue adhesion for wound closure. Crosslinking is a necessary step in hydrogel formation, converting soluble polymers into more stable polymer networks with high water content. This stabilisation can be achieved through a variety of approaches including physical crosslinking such as in freeze-thaw processes [1], formation of ionic complexes such as in calcium crosslinked alginate [2], and self-assembly of peptides. However, covalent crosslinking in physiological conditions is the preferred approach for fabrication of biomedical hydrogels with stable crosslinks and robust mechanical properties [3–8]. In particular, enzymatic crosslinking [9–11] is favoured due to the mild physiological conditions under which the reactions occur.

**Fig. 1:**
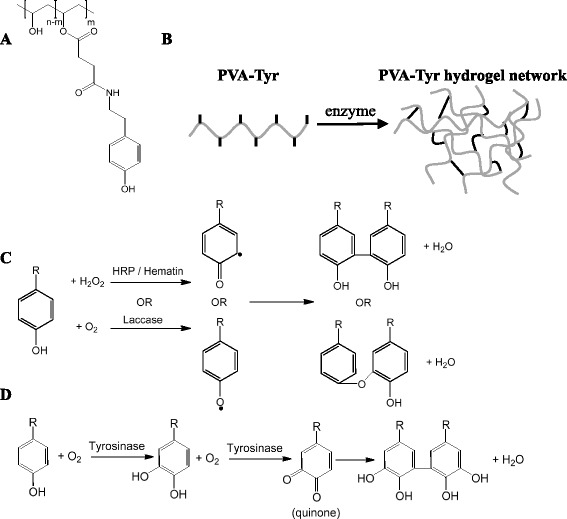
Schematic of PVA-Tyr macromer (*n* = 364, m = 7) (**a**) and polymerization of PVA-Tyr hydrogel network (**b**). Mechanisms of enzymatic oxidative crosslinking of tyramine residues with HRP or hematin and H_2_O_2_ or laccase with O_2_ (**c**), or tyrosinase with O_2_ (**d**)

The objective of this research was to provide a systematic evaluation of enzymatically-mediated covalent crosslinking of phenolic residues in a single polymer system. Phenol crosslinking systems provide advantages over many others, since proteins with tyrosine residues can be incorporated without prior chemical modification. As a result, many natural polymers such as gelatin [12–15] and silk [16], which contain tyrosine residues, can easily be formed into hydrogels with or without synthetic components. To further exploit this bioinspired crosslinking mechanism, many researchers have modified polymers such as dextran [17], hyaluronic acid [11, 18, 19], poly (ethylene glycol) (PEG) [20, 21] and poly (vinyl alcohol) (PVA) [12, 13] with phenolic structures such as tyramine (Fig. 1a, b) [12, 13], hydroxyphenyl propionic acid [20], and 4-hydroxyphenyl acetic acid [10]. However, much of the research conducted is on a limited range of proteins or tyramine modified polymers with only a single crosslinking initiator chemistry explored. This research provides the first insight into the comparative efficacy of four enzyme crosslinking systems, horseradish peroxidase (HRP), hematin, laccase and tyrosinase towards cell encapsulation applications.

HRP is one of the most commonly used enzymes to crosslink phenol residues. HRP is normally combined with the oxidant hydrogen peroxide (H_2_O_2_), where HRP extracts oxygen from the peroxide leading to a change in the oxidation state of its heme group. The HRP reaction can be simply summarized in the following equation: 2RH + H_2_O_2_ → 2R^•^ + 2H_2_O where RH represents the phenol and R^•^ represents the free radical formed (Fig. 1c). The combination of HRP and H_2_O_2_ in the presence of phenolic hydroxyl groups enables crosslinking of the aromatic ring by C-C and C-O coupling, and ultimately leads to hydrogel formation [9–11]. A suggested alternative to using HRP is hematin, which is an enzyme-like molecule that is clinically approved for the management of porphyria attacks [22, 23]. Hematin is an iron-containing porphyrin, with a Fe (III) compound structure similar to the prosthetic iron protoporphyrin IX found in HRP. Similarly to HRP, hematin can be used for the oxidative crosslinking of phenol compounds in the presence of H_2_O_2_ [22–24]. Some oxidoreductase enzymes, such as laccase and tyrosinases do not require H_2_O_2_, but instead use molecular oxygen to crosslink phenol residues. Laccase interacts with molecular oxygen and forms a tightly bound H_2_O_2_, which is then used to form di-phenol crosslinks [25]. Tyrosinase is an enzyme widely found in animals and plants and is responsible for the production of various types of melanin in animal skin and blackening of fruits [26, 27]. Tyrosinase crosslinks phenol residues in a two-step process that is different than the radically-mediated HRP, hematin, and laccase enzymes (Fig. 1d). The key mechanism of the oxidative tyrosinase reaction is the conversion of phenol into catechol by adding an additional hydroxyl group on the aromatic ring using molecular oxygen. Further oxidation of catechol generates a reactive quinone, which readily forms a covalent bond with other quinones [27].

Many of the studies using oxidative enzyme crosslinkers to crosslink phenols use protein or polysaccharide-based hydrogels or biological-synthetic hybrid hydrogels. However proteins and polysaccharides can act as antioxidants, obscuring the effects of the initiating system on cells during encapsulation [13, 21]. Therefore, when evaluating initiators for oxidative, phenol crosslinking the selection of a “blank slate” hydrogel that does not interfere with the initiating system is advantageous. PVA modified with tyramine (PVA-Tyr) groups is an ideal candidate because PVA is a hydrophilic, cytocompatible synthetic polymer for medical applications that is relatively bioinert. As a synthetic polymer, PVA does not natively interact with cells and therefore provides a direct platform for observing the effects of oxidative crosslinking systems on hydrogel formation and cell encapsulation.

The aim of this work was a comparative study that illuminates both the advantages and challenges of various enzyme initiators used to crosslink phenol containing hydrogels using one hydrogel platform, based on a purely synthetic, “blank slate” material. Specifically we wanted to 1) characterize variations in material properties as a function of enzyme type and concentration and 2) evaluate the impact of oxidative, enzyme encapsulation on cells after hydrogel crosslinking. Some of the most commonly used enzyme initiating systems, namely HRP/H_2_O_2_, hematin/H_2_O_2_, laccase, and tyrosinase, were selected to crosslink PVA-Tyr hydrogels. Kinetics were spectroscopically monitored to demonstrate how different concentrations of enzymes and oxidants determine the reaction rate and the final hydrogel quality (i.e., mass loss, swelling, and modulus). Fibroblasts were encapsulated within PVA-Tyr hydrogels using each enzyme initiating system to show the impact of oxidative crosslinking on long-term cell viability and proliferation. Overall, this study demonstrates that a thorough evaluation of enzyme initiators should be performed, especially when using hydrogels crosslinked with oxidants, as the initiator will impact the quality of the hydrogel formed and cellular proliferation, which is critical for biomedical applications.

## Methods

### Materials

Poly (vinyl alcohol) (PVA) (13–23 kDa, 98 % hydrolysed), succinic anhydride, triethylamine, 1,3-dicyclohexylcarbodiimide (DCC), N-hydroxysuccinimide (NHS), tyramine, molecular sieves (4 Å), dialysis tubing (10 kDa molecular weight cut-off), Dulbecco’s Phosphate Buffered Saline (PBS), horseradish peroxidase type VI, hematin porcine, hydrogen peroxide, laccase from trametes versicolor, tyrosinase from mushroom, Dulbecco’s Modified Eagle’s Medium (DMEM), Penicillin-Streptomycin, Trypsin-EDTA Solution 1X, Calcein-AM, Propidium Iodide, Dithiothreitol (DTT), 2-[N-morpholino] ethanesulfonic acid (MES), Tris base, sodium dodecyl sulfate (SDS), ethylenediaminetetraacetic acid (EDTA), phosphoric acid and ammonium sulfate were purchased from Sigma-Aldrich. Fetal bovine serum (FBS) was purchased from Moregate Biotech. Dimethyl sulfoxide (DMSO) dried over 4 Å molecular sieves, ethanol, methanol, and acetone were bought from Ajax Chemicals. Hydrogel disc molds were made from silicone sheets (Silastic®Sheeting, reinforced medical grade silicone rubber, Dow Corning). MTS reagent (CellTiter 96® AQueous One Solution Cell Proliferation Assay) was purchased from Promega. Protein molecular weight markers (Precision Plus Protein™ All Blue Prestained Protein Standards) were purchased from BioRad. Carboxy-H_2_DFFDA, Bis-Tris NuPAGE® SDS-PAGE gels, and Coomassie Blue G250 were purchased from ThermoFisher Scientific.

### Polymer synthesis

PVA-tyramine was synthesized in a two-step reaction as described previously [12]. Briefly, PVA was dissolved in dry DMSO at 60 °C under nitrogen purging and succinic anhydride and triethylamine were added and stirred for 24 h. The carboxylated PVA was precipitated in a 10-fold excess of ethanol and then dialyzed against water prior to lyophilization. Dried carboxylated PVA was dissolved in dry DMSO at 60 °C, and nitrogen purged. The solution was cooled to room temperature and DCC and NHS were added and allowed to react for 24 h, followed by the addition of tyramine for another 24 h. PVA-tyramine was precipitated in 10-fold excess of acetone, re-dissolved in water, and vacuum filtered to remove the dicyclohexylurea byproduct followed by dialysis against water and lyophilization. The PVA-tyramine used in this article was characterized to be 2 % tyraminated (7 tyramine per PVA chain) using ^1^H NMR (300 MHz Bruker Advance DPX-300 spectrometer in D_2_O).

### PVA-tyramine hydrogel formation

PVA-tyramine (5 % w/w) was dissolved in PBS at 80 °C. Upon complete dissolution, the polymer solution was cooled to room temperature and the initiators were added to the solution under gentle vortexing to ensure homogenous gelation. Initiators used were HRP (0–0.5 U/mL) with H_2_O_2_ (0–12 mM), hematin (0–0.08 % w/w) with H_2_O_2_ (0–12 mM), laccase (0–25 U/mL) and tyrosinase (0–2000 U/mL). The macromer solution was then placed into silicone molds (1 mm height x 6 mm diameter) between silicone sheeting (0.1 mm thick). The samples were then placed at 37 °C in a humidified incubator (37 °C and 5 % CO_2_) to gel for 4 h.

### Crosslinking kinetics

Kinetics of oxidative crosslinking were measured by UV-vis spectroscopy. PVA-tyramine hydrogel precursor solutions were formulated as described above and 50 μL of solution was added to the bottom of 96-well plates. UV-vis spectra were recorded on a BMG LABTCH SPECTROstar Nano spectrophotometer for up to 6 h during gelation at 37 °C. Plates were covered with an adhesive plate cover to minimize evaporation.

### Hydrogel swelling and mass loss

Directly after hydrogel polymerization for 4 h, all samples were weighed for the initial wet mass (m_i_) and three samples per study were immediately lyophilised to obtain the initial macromer fraction (m_i,d_). Hydrogel samples were then submerged in a sink of PBS and incubated at 37 °C. Samples were removed from the incubator after 2 days, blotted dry and weighed (m_s_). The swollen samples were then lyophilized and weighed again (m_d_). The mass swelling ratio (q) and mass loss (sol fraction) were calculated as follows:

$$ \begin{array}{l}q=\frac{m_s}{m_d}\hfill \\ {} mass\kern0.5em  loss\%=\frac{m_{i,d}-{m}_d}{m_d}\times 100\%\hfill \end{array} $$

### Rheometry

Fully formed hydrogel samples (4 h gelation) were swollen in a sink of PBS and incubated at 37 °C for two days prior to forming rheological measurements. A Kinexus Pro rheometer from Malvern was used to obtain the magnitude of the (complex) dynamic shear modulus (|G*|) at an oscillation frequency of 1 Hz and a strain of 0.5 % at room temperature (21 °C). Parallel plate geometry with a diameter of 20 mm and a sample gap of 1.0 mm was used. All experiments were performed within the linear viscoelastic region.

### Cell viability and proliferation following encapsulation

L929 murine dermal fibroblasts were trypsinized and resuspended in PBS using aseptic technique. The enzymes were added with gentle mixing to separate sterile macromer solutions at final concentrations of 0.2 U/mL HRP with 6 mM H_2_O_2_, 15 U/mL laccase, or 750 U/mL tyrosinase. The cell suspension was then added to the macromer solution to give a final density of 1x10^6^ cells/ml, of which 50 μL was added to the bottom of wells in a 96-well plate (6 mm diameter). The samples were immediately then placed in a humidified incubator (37 °C and 5 % CO2) and rotated for 4 h at 4 rpm (modified MACSmix Rotator) to ensure homogenous cell suspension during polymerization. The samples were then immersed in media (DMEM, 10 % v/v FBS, 1 % v/v Penicillin-Streptomycin) and cultured for up to 7 days.

Cell viability within gels was determined over one week of culture qualitatively via a Live-dead assay and quantitatively via the MTS assay. At 0 and 7 days post-encapsulation, the samples were stained with 1 μg/ml of Calcein-AM and 1 μg/ml Propidium Iodide in PBS. After 10 min incubation with the stains, the gels were imaged using a confocal microscope (Leica, DM LFSA) while hydrated. After 0, 1, 3, and 7 days cell proliferation was assessed by adding MTS reagent (20 μL) to the cell cultures for 4 h prior to measuring the absorbance at 490 nm. The quantity of formazan product as measured by the amount of 490 nm absorbance is directly proportional to the number of living cells in culture.

### Intracellular ROS production

The cell-permeant dye, carboxy-H_2_DFFDA was used to quantify and visualize intracellular reactive oxygen species (ROS) due to H_2_O_2_ and encapsulation with enzyme initiators, respectively. To measure cell generated ROS by 0–12 mM H_2_O_2_, fibroblasts in suspension culture were incubated with 10 μM carboxy-H2DFFDA for 20 min to allow for its transport into cells and subsequent cleavage of the diacetate followed by several rinses in PBS via centrifugation. Cells (1x10^6^ cells/mL) were resuspended in PBS with 0–12 mM H_2_O_2_ and transferred to 96-well plates (80 μl/well). The plate was placed in a humidified incubator (37 °C and 5 % CO2) and assayed on a Tecan Infinite F200 plate reader at 485 nm excitation 535 nm emission.

### Cell proliferation in the presence of H_2_O_2_

L929 murine dermal fibroblasts were trypsinized, resuspended in PBS containing 0–12 mM H_2_O_2_ and placed in a humidified incubator (37 °C and 5 % CO2). After 4 h cell culture media was added to the wells and cells were then cultured for up to a week. Cell proliferation was assessed by adding MTS reagent to the cell cultures for 4 h prior to measuring the absorbance at 490 nm.

### Statistical analysis

A one-way or two-way analysis of variance (ANOVA) was performed to compare multiple conditions with Tukey’s post-hoc analysis. Significance was tested using GraphPad Prism 6 (GraphPad Software) and results of *p* < 0.05 were considered significant. Experiments were performed in triplicate and experiments were repeated three times. Quantitative data are expressed as mean with error bars representing standard deviation (mean ± SD).

## Results and discussion

### Gelation, swelling, mass loss, and mechanical properties of phenol-modified hydrogels crosslinked with various enzyme initiators

Synthetic hydrogels based on PVA modified with the phenol molecule tyramine (Fig. 1a) were successfully crosslinked using the initiators HRP/H_2_O_2_, hematin/H_2_O_2_, laccase and tyrosinase under physiological conditions (37 °C, pH 7.4) (Fig. 1b). For all of the different initiating systems examined, there is a significant increase in absorbance around 325 nm during the crosslinking process, indicating the formation of dityrosine crosslinks [26]. Therefore, this increase in absorbance at 325 nm is monitored over the crosslinking period as a measure of the polymerisation kinetics, where complete gelation is defined as the time when no further changes in absorbance is observed.

**Fig. 2:**
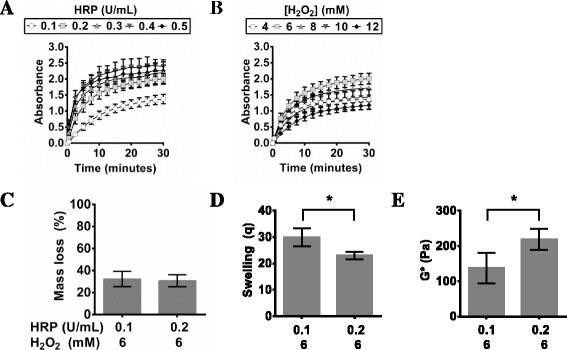
The change in absorbance of PVA-Tyr during HRP/H_2_O_2_–mediated polymerization in PBS at 37 °C at 325 nm via UV-vis spectrophotometry over time with **a** varying HRP (U/mL) and constant H_2_O_2_ (6 mM) and (**b**) **a** constant HRP (0.2 U/mL) and varying H_2_O_2_ (mM). After gelation, hydrogels were swollen and the **c** mass loss, **d** swelling, and **e** dynamic shear modulus (G*) were measured after 48 h at 37 °C. Significance (*p* < 0.05) is denoted in bar graphs C-E with an asterisk (*)

Initially, the PVA-Tyr macromer gelation was monitored over a range of HRP and H_2_O_2_ concentrations, similar to that used previously for enzyme crosslinked hydrogels [20, 21]. It has been suggested that for this enzyme-mediated gelation process, HRP controls the kinetics of gelation [9, 20, 21]. Hence the time to complete gelation of 5 % w/w PVA-Tyr was firstly monitored by varying the HRP concentration from 0.1 to 0.5 U/mL at a constant 6 mM H_2_O_2_. As expected, the gelation time decreased from 30 to 10 min with increasing HRP concentrations (Fig. 2a, Table 1). Gelation rate is an important factor to consider in hydrogel development, since there is a fine balance between having efficient gelation when encapsulating cells, versus having gelation that is too rapid and is impractical for the researcher or physician when delivering the cell-hydrogel mixtures [20]. Subsequently, a HRP concentration of 0.2 U/mL was selected since 0.1 U/mL resulted in significantly lower polymerization and rapid gelation was observed at concentrations higher than 0.2 U/mL. The gelation of 5 % w/w PVA-Tyr was then monitored as a function of H_2_O_2_ concentration at constant 0.2 U/mL HRP. As the H_2_O_2_ concentration increased from 4 mM to 6 mM the absorbance at complete gelation significantly increased, suggesting a more crosslinked hydrogel (Fig. 2b). However as the H_2_O_2_ concentration increased above 6 mM, the absorbance at final gelation decreased, indicating less crosslinking likely due to the inhibitory effect of excess H_2_O_2_ on HRP activity [20, 25]. Hydrogen peroxide serves as oxidant and therefore is essential for catalytic action. However, in the presence of an excess of H_2_O_2_ the heme reacts with another equivalent of H_2_O_2_ instead of returning to the resting state which leads to irreversible enzyme inactivation [25, 28]. As these results confirmed the hypothesis that HRP controls the reaction kinetics and a concentration of H_2_O_2_ greater than 6 mM impairs the crosslinking reaction, the H_2_O_2_ concentration was kept constant at 6 mM for further studies.

**Table 1:** Physical properties of PVA-Tyr hudrogels crosslinked via varying enzyme types and concentrations

Enzyme			Oxidant			Time to gel (min)^a^	Final absorbance at gelation (325 nm)^b^	Sol fraction (% mass loss at 48 h)	Swelling (q, at 48 h)	Crosslinking density (ρ_x_, mmol/L)^c^	Dynamic shear modulus (G*, Pa)
Name	Concentration	Unit	Name	Concentration	Unit						
HRP	0.1	U/mL	H_2_O_2_	6	mM	20	1.4 ± 0.2	32 ± 7 %	29 ± 3	2.2	138 ± 43
	0.2			6		15	2.0 ± 0.2	31 ± 5 %	23 ± 1	3.8	219 ± 30
	0.2			12		20	1.2 ± 0.1	36 ± 7 %	33 ± 5	1.6	89 ± 24
Hematin	0.01	% (w/w)	H_2_O_2_	6	mM	60	0.3 ± 0.0	76 ± 8 %	168 ± 36	0.1	5 ± 1
	0.06			6		15	0.7 ± 0.2	63 ± 10 %	121 ± 25	0.1	11 ± 3
	0.06			12		30	1.1 ± 0.1	49 ± 11 %	84 ± 16	0.2	38 ± 5
Laccase	5	U/mL	O_2_			360+	2.4 ± 0.2	33 ± 6 %	26 ± 4	2.8	97 ± 34
	15					240	2.9 ± 0.1	24 ± 10 %	21 ± 3	4.6	145 ± 22
Tyrosinase	500	U/mL	O_2_			150	1.7 ± 0.1	26 ± 5 %	44 ± 7	0.9	145 ± 49
	750					150	2.0 ± 0.2	20 ± 8 %	35 ± 10	1.4	169 ± 56

^a^Gelation time was determined as the time when there was no further significant increase in absorbance

^b^The absorbance after the hydrogel had completed gelation as determined by the time to gel

^c^Crosslinking density was determined from swelling (q) at 48 h using equations derived from Flory and Rehner [30]

PVA-Tyr hydrogels fabricated using 0.1 and 0.2 U/mL HRP (6 mM H_2_O_2_) had significantly different final absorbance values and were further compared in terms of the physico-mechanical properties. Soluble (sol) fraction or mass loss after initial swelling, swelling and modulus of fully gelled hydrogels are shown in Fig. 2c, d, e, respectively. The mass loss within PVA-Tyr hydrogels crosslinked with HRP/H_2_O_2_ as an initiation system have ~30 % mass loss (Fig. 2c), similar to the initial mass loss within other hydrogel studies [29]. As the HRP concentration was increased (0.1 to 0.2 U HRP/mL, 6 mM H_2_O_2_) swelling significantly decreased, which corresponds with the varying degrees of gelation demonstrated using spectrophotometry (Fig. 2d). The final dynamic shear modulus (G*, Fig. 2e) follows the opposite trend, where a decrease in swelling of HRP/H_2_O_2_ crosslinked PVA-Tyr gels results in a significant increase in mechanical properties and vice versa. This result agrees with the literature where an increase in crosslinking density is reflected by a decrease in equilibrium water content (swelling). This decrease in the equilibrium water content (swelling) results in an increase in the crosslinking density (ρ_x_) and an increase in the mechanical properties, such as shear modulus [30]. Overall, it can be seen that the HRP/H_2_O_2_ initiating system can be used to effectively crosslink phenol-modified macromers and that the concentration of HRP is critical to determining the rate and degree of polymerization.

**Fig. 3:**
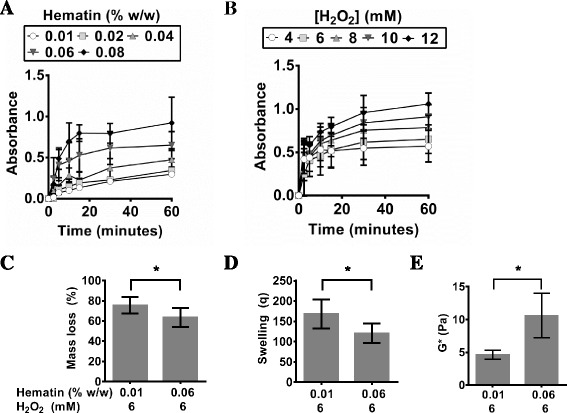
The change in absorbance of PVA-Tyr during hematin/H_2_O_2_–mediated polymerization in PBS at 37 °C at 325 nm via UV-vis spectrophotometry over time with **a** varying hematin (% w/w) and constant H_2_O_2_ (6 mM) or **b** constant hematin (0.06 % w/w) and varying H_2_O_2_ (mM). After gelation, hydrogels were swollen and the **c** mass loss, **d** swelling, and **e** dynamic shear modulus (G*) were measured after 48 h at 37 °C. Data in **a** and **b** were presented to 60 min because there was no further absorbance increase after this time. Significance (*p* < 0.05) is denoted in bar graphs C-E with an asterisk (*)

Next, the crosslinking kinetics of PVA-Tyr hydrogels fabricated using hematin/H_2_O_2_ were examined in a similar fashion. Hematin has been suggested as an alternative to HRP, as it also has a heme structure and requires the use of an oxidant such as H_2_O_2_ to crosslink phenol residues as shown in Fig. 1c. As the concentration of hematin increased from 0.01 to 0.08 %w/w at constant 6 mM H_2_O_2_, the final absorbance at 325 nm significantly increased, demonstrating a higher degree of phenol crosslinking (see Fig. 3a). Similarly as the H_2_O_2_ concentration was increased from 4 to 12 mM at constant 0.06 % w/w hematin, the final degree of polymerization also increased (see Fig. 3b). Absorbance was monitored for 360 min for all hematin/H2O2 concentrations, however absorbance increases were not significant after 60 min and so data was presented to 60 min to emphasize the early polymerization kinetics (Fig. 3a, b). When keeping the H_2_O_2_ concentration constant at 6 mM similar to previous study done with HRP, as the hematin of concentration increased, there was a significant decrease in the initial mass loss (Fig. 3c). This is because a higher degree of polymerization implies that more PVA polymer chains have been crosslinked into the hydrogel, and thus there is lower initial mass loss due to uncrosslinked PVA-Tyr macromers diffusing out. This demonstrates that a higher hematin concentration leads to higher crosslinking efficiency of the phenol residues, as has been previously suggested [22]. This higher crosslinking efficiency results in a significant decrease in swelling and an significant increase in mechanics (Fig. 3d, e). At a similar oxidant concentration (6 mM H_2_O_2_), hematin (Fig. 3) crosslinks PVA-Tyr hydrogels significantly less efficiently than HRP (Fig. 2), as shown by lower final absorbance, higher mass loss, and lower shear modulus respectively. This difference between hematin and HRP may be in part due to the low solubility of hematin, which significantly hinders its widespread use [22, 23, 31]. Previous studies has shown that hematin had to be dissolved in alkaline buffer and when the solution pH was reduced to 7.4, crude aggregates are formed [22]. Therefore, the hematin concentration examined in this study was set to a maximum of 0.08 % w/w to ensure its complete dissolution [22]. It has also been shown that hematin requires significantly higher amounts of H_2_O_2_ when compared to HRP to reach similar activity levels. For example, one study evaluated the activity of hematin and HRP for crosslinking phenol molecules and found that 50 mM of H_2_O_2_ was required for hematin as compared to HRP which only needed 1 mM H_2_O_2_ to get similar level of activity [22]. In terms of using hematin/H_2_O_2_ for crosslinking PVA-Tyr hydrogels in the presence of cells, although it was hypothesized that higher degree of crosslinking can be achieved with elevated levels of H_2_O_2_, it was not conducted in this study as high concentrations of H_2_O_2_ are cytotoxic to cells [21].

**Fig. 4:**
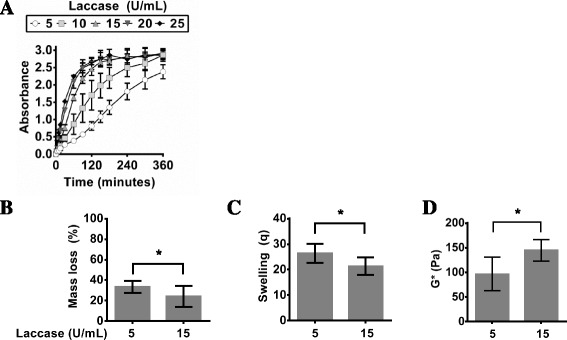
The change in absorbance of PVA-Tyr during laccase-mediated polymerization in PBS at 37 °C at 325 nm via UV-vis spectrophotometry over time (**a**). After gelation, hydrogels were swollen and the **b** mass loss, **c** swelling, and **d** dynamic shear modulus (G*) were measured after 48 h at 37 °C. Significance (*p* < 0.05) is denoted in bar graphs B-D with an asterisk (*)

Although it was shown in this study that HRP/H_2_O_2_ and hematin/ H_2_O_2_ can successfully fabricate PVA-Tyr hydrogels, the use of H_2_O_2_ might still raise issues for *in situ* crosslinking of cell-laden hydrogels as it may cause tissue damage and lipid peroxidation [32]. Therefore, other enzymes such as laccase and tyrosinase that are able to crosslink phenol moieties without the need of oxidants such as H_2_O_2_ are of interest. At low concentration of laccase (5 and 10 U/ml), all the PVA-Tyr macromers had not fully gelled after 6 h as suggested by the lack of plateau of the absorbance curve (Fig. 4a). However, at concentrations higher than 15 U/ml, it was shown that all conditions resulted in similar polymerization profiles, suggesting that the maximum efficiency of laccase had been reached at 15 U/mL (Fig. 4a). Above 15 U laccase/mL, PVA-Tyr macromers took ~2.5 h to reach full polymerization. Although laccase does not require H_2_O_2_ to initiate phenol crosslinking, it uses molecular oxygen as an oxidant to initiate the crosslinking of phenol residues (Fig. 1c). This is advantageous because it eliminates the need for H_2_O_2_ and thus reduces the number of reagents required for hydrogel mixing. However, this means that hydrogel gelation in the presence of laccase is limited by the diffusion of oxygen. An increase in the laccase concentration from 5 to 15 U/mL led to a higher degree of gelation as suggested by the significantly lower initial mass loss, significantly lower swelling and significantly higher dynamic shear modulus at 15 U/mL (Fig. 4b, c & d). Analysis of polymerization of PVA-Tyr with laccase demonstrates that although gelation is slower than that of HRP/H_2_O_2_ or hematin/H_2_O_2_, laccase-mediated polymerization can result in highly crosslinked hydrogel networks as suggested by analysis of the material properties.

**Fig. 5:**
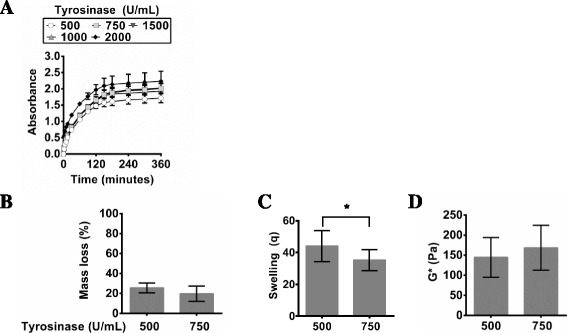
The change in absorbance of PVA-Tyr during tyrosinase-mediated polymerization in PBS at 37 °C at 325 nm via UV-vis spectrophotometry over time (**a**). After gelation, hydrogels were swollen and the **b** mass loss, **c** swelling, and **d** dynamic shear modulus (G*) were measured after 48 h at 37 °C. Significance (*p* < 0.05) is denoted in bar graphs B-D with an asterisk (*)

Another enzyme examined in this study is tyrosinase, which like laccase, does not require any oxidants to crosslink phenol groups. However, tyrosinase-mediated gelation of phenols occurs via a vastly different mechanism than HRP/H_2_O_2_, hematin/H_2_O_2_, and laccase (Fig. 1d) [26, 27, 33]. Although the end result of tyrosinase-mediated polymerization is similarly a crosslinked hydrogel, it is important to monitor the gelation behaviour and material properties to probe for any variations due to the different crosslinking mechanism. Increasing tyrosinase concentration from 500 to 750 U/mL significantly increases the final degree of polymerization (Fig. 5a). At concentrations above 750 U/mL there was no significant difference in the absorbance after 2.5 h of gelation. The resulting material properties of tyrosinase-crosslinked PVA-Tyr that had been polymerized for 4 h showed that increasing the tyrosinase concentration led to a decrease in the average initial mass loss (Fig. 5b). Similarly, there was a significant decrease in swelling which corresponded to an increase in the dynamic shear modulus as the tyrosinase concentration was increased from 500 to 750 U/mL (Fig. 5c & d). Similarly to laccase, tyrosinase-mediated crosslinking of phenols is limited by the diffusion of oxygen (Fig. 1c, d). The diffusion coefficient of oxygen in water is 2.9x10^-5^ cm^2^/s, and therefore oxygen will diffuse through the 1 mm thick hydrogel samples in approximately 6 min, if the hydrogels (95 % water) are approximated as water [34]. However, oxygen diffusion is also impacted by viscosity, which increases during gelation, and it has been shown that fully polymerized hydrogels have lower oxygen diffusivity than water [34]. Moreover, the rate of phenol crosslinking will also be affected by the enzyme activity, which is dependent on temperature, pH, enzyme concentration, substrate concentration, and dissolved oxygen content. The dissolved oxygen content in water at 37 °C is only 6.7 mg/L, which implies that there was approximately 60 times less oxygen molecules than phenol molecules in the macromer solution when the enzyme was added (time = 0) [34]. Therefore, it is not surprising that the laccase- and tyrosinase-mediated crosslinking of PVA-Tyr had slower reaction kinetics than the HRP and hematin enzyme initiating systems that initially contained a sufficient concentration of oxidant (H_2_O_2_). PVA-Tyr hydrogels crosslinked using HRP or hematin with H_2_O_2_ polymerized in under 30 min, whereas gels crosslinked with laccase or tyrosinase polymerized in a minimum of 2.5 h to over 6 h.

Overall, it can be seen that all enzymes and enzyme-like initiators evaluated were able to crosslink phenol-modified macromers into hydrogels. Gelation monitored using spectroscopy showed that varying the initiator/oxidant type and concentration led to final polymerization times ranging from 10 min to over 6 h under physiological conditions (37 °C, pH 7.4). A higher enzyme and oxidant concentration generally led to a higher degree of polymerization, with the exception of HRP where too much oxidant is known to reduce reaction efficiency [20, 25]. The sol fraction was evaluated versus absorbance, as a relative measure of crosslinking efficiency of all four enzyme and enzyme-like initiators. A higher PVA-Tyr hydrogel absorbance corresponded to lower hydrogel mass loss which suggests a larger fraction of PVA chains being crosslinked into the gel up until an absorbance of ~1.2 (Additional file 1: Figure S1). It is hypothesized all PVA chains functionalized with tyramine continued to further crosslink as the absorbance increased from 1.2 to 2.9 as suggested by a decrease in swelling (Table 1), however all of these samples had ~25 % mass loss because of unfunctionalized PVA that came out as the sol fraction. HRP and hematin with H_2_O_2_ as an oxidant both led to rapid gelation of PVA-Tyr hydrogels within 30 min, whereas laccase and tyrosinase which use dissolved O_2_ as an oxidant gelled significantly slower (2.5+ hours). Although the use of H_2_O_2_ as an oxidant leads to rapid polymerization which is advantageous for *in situ* clinical gelation, H_2_O_2_ is also known to reduce cell proliferation and be cytotoxic at high concentrations which leads to questions about its use for cell encapsulation and/or for wound closure [21, 35].

### Cell viability and proliferation after encapsulation in phenol-modified hydrogels crosslinked with various enzyme initiators

Although HRP/H_2_O_2_, hematin/H_2_O_2_, laccase and tyrosinase can all form robust hydrogels from phenol-modified polymers, it is also critical for biomedical applications to evaluate the impact of the enzyme-mediated crosslinking process on cells. Based on the hydrogel material properties, enzyme initiators that resulted in the highest degree of polymerization were selected for cell encapsulation (i.e. sol fraction ~25 %, swelling q ~ 25, G* ~ 180 Pa). Hematin/H_2_O_2_ crosslinked gels did not reach the same material properties as gels formed from the other initiators, and was not evaluated for cell encapsulation because of the high H_2_O_2_ concentrations required. PVA-Tyr hydrogels crosslinked for 4 h with HRP/H_2_O_2_ (0.2 U/mL/6 mM), laccase (15 U/mL), and tyrosinase (750 U/mL) had similar swelling and mechanical properties, and thus a similar crosslinking density [30]. Having a similar crosslinking density will allow for direct comparison between the fully formed hydrogels because it implies that oxygen and nutrient diffusion will be similar within all hydrogels.

**Fig. 6:**
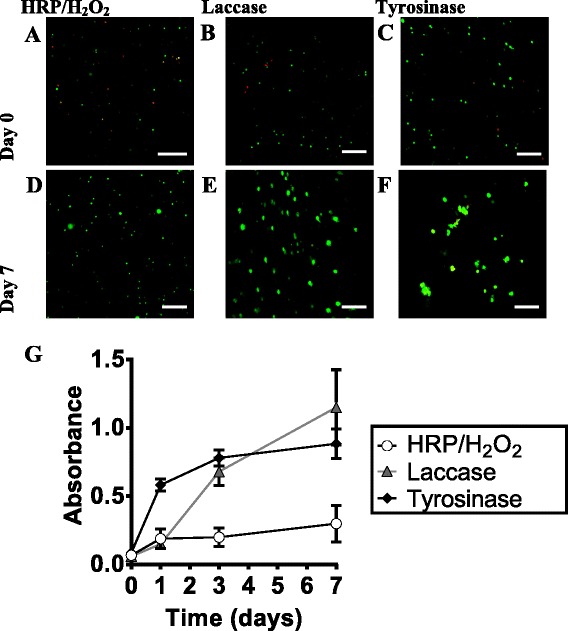
Representative confocal microscopy images of fibroblast viability within in PVA–Tyr hydrogels immediately after 4 h of gelation (**a**, **b**, **c**) or after 7 days of culture (**d**, **e**, **f**). PVA–Tyr hydrogels were polymerized with different enzyme initiators; A,D) HRP (0.2 U/mL) with H_2_O_2_ (6 mM), **b**, **e**) laccase (15 U/mL), and **c**, **f**) tyrosinase (750 U/mL). Live cells fluoresce green and dead cells fluoresce red. Scale bars represent 200 μm. **g** Fibroblast cell number was determined quantitatively over 7 days within the PVA-Tyr hydrogels by the MTS assay (absorbance at 490 nm). The lines between data points are meant to guide the eye in observing the trends of the data

All enzyme initiators led to the formation of solid, PVA-Tyr hydrogels in the presence of fibroblast cells. Immediately after encapsulation with the three different initiating systems there was a high degree of cell viability (>80 %) in the hydrogels, as measured by the Live-dead assay (Fig. 6a–c). However when the images were analysed semi-quantitatively, the hydrogels polymerized with HRP/H_2_O_2_ had significantly lower viability (82 ± 5 %), as compared with hydrogels that used oxygen as an oxidant (laccase: 92 ± 6 %, tyrosinase: 89 ± 3 %). After 7 days, there was no significant difference between the viability (>90 %) in hydrogels polymerized with all three different initiators (Fig. 6d–f). However, it could be seen that after 7 days, there were larger cell clumps in hydrogels polymerized with laccase (Fig. 6e) and tyrosinase (Fig. 6f), whereas cells polymerized using HRP/H_2_O_2_ did not form as large of clumps (Fig. 6d). This data is reflected with the MTS assay. This assay measures the reduction of MTS tetrazolium by NAD (P)H-dependent dehydrogenase enzymes in metabolically active cells, and thus is used as a quantitative measure of cell viability and proliferation. In each enzyme initiating system, cells proliferated over the 7 days of culture within the phenol-modified hydrogels (Fig. 6g). However, the hydrogels polymerized with HRP which utilises the oxidant H_2_O_2_ had significantly lower rates of proliferation between days 1 and 7 than those polymerized with laccase or tyrosinase. This suggests that the enzyme initiating system oxidant is likely what impacts cell proliferation between days 1 and 7. PVA-Tyr hydrogels polymerized with laccase had a similar number of cells as those polymerized with HRP/H_2_O_2_ at day 1, however, cell proliferation recovered in these gels between day 1–7. Although laccase uses molecular oxygen as an oxidant, it converts the oxygen to hydrogen peroxide, which it then uses similarly to HRP and hematin to generate phenoxy radicals that crosslink phenol residues [36]. The recovery in cell proliferation in laccase may be because H_2_O_2_ produced by laccase is bound very tightly within the enzyme, unlike HRP where the H_2_O_2_ has to be dissolved in solution [25]. Excessive H_2_O_2_ is known to lead to oxidative stress in cells and disease [37]. When fibroblasts were incubated with hydrogen peroxide at concentrations similar to those used for encapsulation (0–12 mM H_2_O_2_) there was an upregulation of intracellular reactive oxygen species (ROS), which increased with H_2_O_2_ concentration and was evident after as little as 10 min of incubation (Additional file 1: Figure S2). This shows that extracellular H_2_O_2_ can cross the cell membrane and lead to intracellular activity, such as ROS generation within the cells. To test the impact of this low concentration of H_2_O_2_ on cell viability and proliferation, fibroblasts were temporarily incubated with varying H_2_O_2_ concentrations and then cultured for a week. Cells plated without H_2_O_2_ had a significant increase in proliferation after day 1 (Additional file 1: Figure S3), which was similar in absorbance to cells encapsulated in PVA-Tyr gels crosslinked with tyrosinase. However at concentrations between 0.2 and 12 mM H_2_O_2_ the cells attached to the plate, but did not significantly proliferate over 7 days of culture (Additional file 1: Figure S3 C, D). Depression of protein synthesis, DNA synthesis, and proliferation has been seen with human fibroblasts incubated with as little as 500 μM H_2_O_2_ [35]. This suggests that even very small amounts of residual H_2_O_2_ during polymerization can have a large impact on cellular behaviour. Moreover, when H_2_O_2_ is used to encapsulate cells within hydrogels at high concentrations it can have cytotoxic effects. In purely synthetic hydrogel based on tetronic hydrogel (a four-armed block copolymer of poly (ethylene oxide) and poly (propylene oxide) it was demonstrated that enzymatically crosslinking using HRP with ~80 mM H_2_O_2_ could lead to the death of cells during encapsulation [21]. These results demonstrate the importance of ensuring rapid gelation (i.e. rapid consumption of H_2_O_2_) and using minimal H_2_O_2_ concentrations. Alternatively, researchers commonly make biosynthetic, hybrid hydrogels whereby proteins, such as gelatin or fibronectin, are incorporated that can be used as antioxidants to protect cells from oxidizing radicals [13, 20]. For example, it was shown that when HRP/H_2_O_2_ were used to crosslink a biosynthetic fibronectin-PEG hydrogel, mesenchymal stem cell viability 1 day after encapsulation was significantly higher than when compared to a purely synthetic PEG hydrogel [20]. As opposed to the enzymatic, radical initiated polymerizations, tyrosinase-mediated polymerization led to a higher metabolic activity within 1 day after encapsulation (Fig. 6g). Over the 7 days of encapsulation cells in PVA-Tyr hydrogels crosslinked with tyrosinase had very similar proliferation behaviour to those cultured in well plates without any H_2_O_2_ (Fig. 6, Additional file 1: Figure S3). Tyrosinase is not a radical-based polymerization and instead generates a reactive quinone to initiate crosslinking. In mammalian tissue tyrosinases initiate the biosynthetic production of melanins by converting the amino acid tyrosine to a quinone that undergoes spontaneous reactions to yield melanin. Since tyrosinase is naturally produced in mammalian tissue its quinone-based crosslinking mechanism may be milder on cells during encapsulation.

## Conclusions

This study provided a single bioinert, hydrogel platform to systematically evaluate four different enzyme, and enzyme-like, initiators for their ability to crosslink phenol-based macromers and the influence of this oxidative crosslinking on cells during encapsulation. These *in situ* forming, enzymatically crosslinked phenol-containing hydrogels can be used for a large variety of biomedical applications including as adhesives for wound closure [23], for drug delivery [9], and for cell encapsulation for engineering tissues such as cartilage [17] and neural [15] tissue. All enzyme initiators evaluated (HRP/H_2_O_2_, hematin/H_2_O_2_, laccase, tyrosinase) can be used to form phenol-crosslinked hydrogels, however gelation rates are dependent on enzyme type, concentration, and the oxidant. HRP or hematin with hydrogen peroxide led to a more rapid PVA-Tyr polymerization because a high oxidant concentration was dissolved within the macromer solution at the onset of crosslinking, but the HRP/H_2_O_2_ initiating system also led to decreased cell survival. Whereas laccase and tyrosinase require oxygen diffusion to crosslink phenol residues and took longer to gel, cells encapsulated in these gels proliferated over time. Overall this study demonstrates that there are many available enzyme and enzyme-like initiators to create injectable, *in situ* phenol-crosslinked hydrogels. However care must be taken when selecting the appropriate enzyme as the oxidoreductase and hematin initiators evaluated in this study require oxidants to polymerize phenol residues, which can affect cells during encapsulation and may alter the long-term therapeutic potential of the cells for tissue engineering applications.

## Additional file

Additional file 1: Additional figures show hydrogel crosslinking efficiency with all four initiators, intracellular ROS during H_2_O_2_ incubation, and fibroblast proliferation after H_2_O_2_ exposure. (DOCX 351 kb)

## Abbreviations

|G*|: (Complex) dynamic shear modulus
ANOVA: Analysis of variance
DCC: 1,3-dicyclohexylcarbodiimide
DMEM: Dulbecco’s modified eagle’s medium
DMSO: Dimethyl sulfoxide
DTT: Dithiothreitol
EDTA: Ethylenediaminetetraacetic acid
FBS: Fetal bovine serum
H_2_O_2_: Hydrogen peroxide
HRP: Horseradish peroxidase
M_d_: Dried sample mass
MES: 2-[N-morpholino]ethanesulfonic acid
M_i_: Initial wet mass
M_i,d_: Initial macromer fraction
M_s_: Swollen sample mass
NHS: N-hydroxysuccinimide
PBS: Dulbecco’s phosphate buffered saline
PEG: Poly(ethylene glycol)
PVA: Poly(vinyl alcohol)
PVA-Tyr: Poly(vinyl alcohol)-tyramine
Q: Mass swelling ratio
ROS: Reactive oxygen species
SDS: Sodium dodecyl sulfate
