# Supplementary material for: A comparative study of enzyme initiators for crosslinking phenol-functionalized hydrogels for cell encapsulation
Source: Biomater Res. 2016 Oct 5;20:30. doi: 10.1186/s40824-016-0077-z (PMC5050849; doi:10.1186/s40824-016-0077-z)
Supplement: Additional file 1: — Additional figures show hydrogel crosslinking efficiency with all four initiators, intracellular ROS during H2O2 incubation, and fibroblast proliferation after H2O2 exposure. (DOCX 351 kb) [file 40824_2016_77_MOESM1_ESM.docx]

Supporting Information

**A comparative study of enzyme initiators for crosslinking phenol-functionalized hydrogels for cell encapsulation**

Justine J. Roberts, Pratibha Naudiyal, Khoon S. Lim, Laura A. Poole-Warren, and Penny J. Martens

**Figure S1.** The sol fraction (mass loss at 48 hours), which indicates of crosslinking efficacy, versus absorbance at final gelation (325 nm) for 5% (w/w) PVA-Tyr hydrogels crosslinked with HRP/H_2_O_2_, hematin/H_2_O_2_, laccase, or tyrosinase. As absorbance increases there is higher incorporation of PVA chains as indicated by absorbance increasing from dityramine bonding.

**Figure S2.** Intracellular ROS generation in fibroblasts as measured by fluorescence using carboxy-H_2_DFFDA. Fibroblasts were suspended in PBS containing varying concentrations of H_2_O_2_ (0-12 mM) and fluorescence was measured over time at 10 minutes (circles) and 4 hours (squares) after the addition of the H_2_O_2_.

**Figure S3.** Fibroblast’s were incubated with 0-12 mM H_2_O_2_ in PBS for 4 hours (the same duration as PVA-Tyr gelation) and then cultured. Cells incubated with no H_2_O_2_ (i.e., in PBS) proliferate significantly, whereas cells incubated with 0.2-12 mM H_2_O_2_ but have no significant proliferation over 7 days. Cell number was determined quantitatively using the MTS assay (absorbance at 490 nm) over 7 days of culture. Images of fibroblasts plated in A) 0 mM H_2_O_2_ after 1 day B) 0 mM H_2_O_2_ after 3 days C) 0.4 mM H_2_O_2_ after 1 day and D) 0.4 mM H_2_O_2_ after 3 days, demonstrating that cells were attached, suggesting cell viability, after being incubated with varying H_2_O_2_ concentrations. Scale bars represent 100 µm.
